# Intermittent high-fat diet: atherosclerosis progression by neutrophil reprogramming

**DOI:** 10.1038/s41392-024-02027-4

**Published:** 2024-11-07

**Authors:** Andrea Herrero-Cervera, Raphael Chevre, Oliver Soehnlein

**Affiliations:** Institute of Experimental Pathology (ExPat), Center for Molecular Biology of Inflammation (ZMBE), University Hospital Münster, University of Münster, Münster, Germany

**Keywords:** Cardiology, Inflammation

A recent study published in *Nature* by Lavillegrand et al. provided insights into how alternating high-fat diet (HFD) feeding reprograms neutrophil production and consequently promotes atheroprogression.^1^ These findings have translational implications, offering insights into the use of dietary patterns as a potential therapeutic strategy to mitigate atherosclerosis.

Atherosclerosis, the underlying pathology of myocardial infarction, ischemic cardiomyopathy, stroke, and peripheral arterial disease, is the leading cause of death worldwide. One of the traditional risk factors for atherosclerosis is hypercholesterolemia, which promotes activation of the bone marrow (BM) and heightened production of neutrophils with increase of neutrophils in the blood stream. However, with the widespread use of lipid-lowering drugs and better control of hypercholesterolemia in patients, attention has shifted to dietary patterns, including yo-yo dieting, also known as weight cycling. Changes in dietary habits are common throughout life, and fluctuations in body weight (as a proxy to altered dietary patterns) are associated with a higher rate of cardiovascular events in patients with a history of coronary artery disease, independent of other cardiovascular risk factors.^2^ However, the mechanisms underlying such association remain ill-defined.

**Fig. 1:**
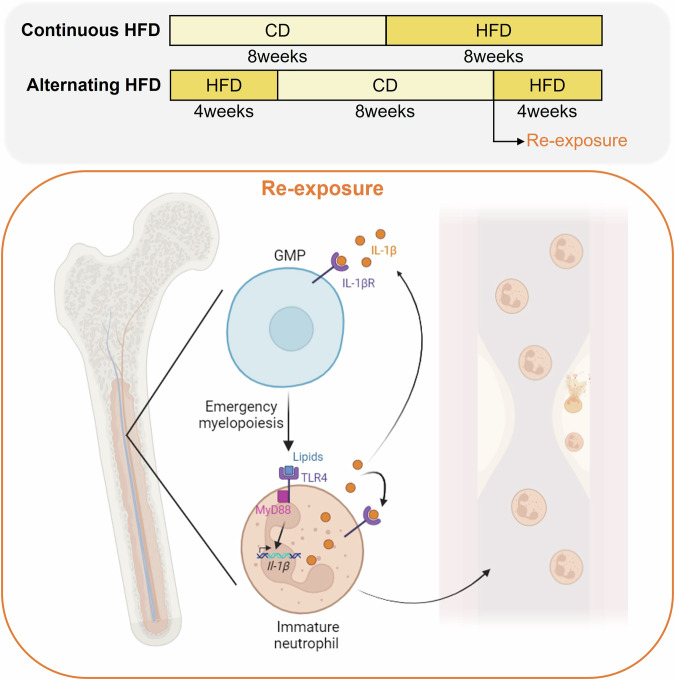
HFD re-exposure promotes neutrophilia through IL-1β signaling and atherosclerosis. Alternating HFD involved feeding with a HFD for 4 weeks, switching to a CD for 8 weeks, and then re-exposure to HFD for an additional 4 weeks. The control group was fed a CD for 8 weeks, followed by continuous exposure to HFD for 8 weeks. Upon re-exposure to HFD, lipids activated the TLR4-MyD88 signaling pathway, leading to IL-1β production by neutrophils. IL-1β bound to its receptor in GMPs, triggering emergency myelopoiesis in the BM, which resulted in a reduction of GMPs and an increase in immature neutrophils. These immature neutrophils were released into circulation, increasing their numbers and infiltrating the atheroma plaque, where they released NETs, contributing to the progression of atherosclerosis. Figure created by BioRender

Recent work investigates in detail how a dietary regimen, alternating between a HFD and chow diet (CD) in mice, influences immunity and atherosclerosis.^1^ Herein, Lavillegrand and colleagues designed a dietary pattern called alternating HFD (switch between HFD and CD feeding) in mice which was compared to continuous HFD feeding (Fig. 1). The mice on alternating HFD exhibited accelerated and more advanced atherosclerosis development compared to those continuously fed HFD. This surprising finding was independent of plasma cholesterol levels, gut microbiota, or responses of adaptive immunity. Yet, RNA sequencing (RNAseq) analyses of the aorta suggested a possible role of myeloid cells, as several pathways related to myeloid homeostasis and oxidative stress were either upregulated or downregulated in the alternating HFD group. Analysis of circulating monocytes and neutrophils showed that both populations followed the same pattern as plasma cholesterol levels—higher during HFD and lower during the chow diet. Importantly, unlike monocytes, re-exposure to HFD dramatically increased the number of circulating neutrophils with an immature phenotype. Additionally, single-cell RNAseq (scRNAseq) identified two neutrophil clusters in the aorta, with one of them exhibiting a pro-inflammatory signature, being enriched during alternating HFD and neutrophils infiltrating the plaque under alternating HFD were more prone to release neutrophil extracellular traps (NETs). Supporting these findings, when neutrophils were depleted in mice on alternating HFD, the pro-atherogenic effects were abolished. Neutrophils are mostly produced in the BM and then released into the circulation. Re-exposure to HFD led to emergency myelopoiesis evidenced by a decrease in granulocyte-monocyte progenitors (GMPs) and an increase in immature neutrophils (Fig. 1). This was accompanied by higher production of interleukin (IL)-1β and IL-6 in the BM. Consistent with these findings, *low-density lipoprotein receptor knockout (Ldlr*^*–/–*^*)* mice transplanted with BM cells previously exposed to HFD developed larger atheroma plaques. Furthermore, toll-like receptor (TLR) 4 and myeloid differentiation (MyD) 88 were shown to mediate emergency myelopoiesis, as mice deficient in these genes did not develop neutrophilia or exhibit high BM *Il-1β* mRNA expression after HFD re-exposure. Moreover, at the GMP level, scRNAseq revealed downregulated expression of *Runx1*, and mice deficient in Runx1 in their GMPs showed neutrophilia and higher IL-1β production by neutrophils in the BM after HFD re-exposure. Finally, to explore the role of IL-1β production in the BM in emergency myelopoiesis and the development of atherosclerosis under alternating HFD, *Ldlr*^*–/–*^ mice were transplanted with *Il-1β*^*–/–*^ BM cells or *Il-1r*^*–/–*^ BM progenitors and myeloid cells. After recovery, the mice were fed either continuous or alternating HFD. In both transplantation models, neutrophilia and atherosclerosis were consistently abolished in the alternating HFD group a finding recapitulated in mice receiving the NLR family pyrin domain containing 3 (NLRP3) inflammasome inhibitor MCC950.

In summary, this study provides a mechanistic foundation for how alternating HFD promotes atherosclerosis. Central to their findings are the alteration of BM output in terms of numbers and quality and the striking lesional neutrophil infiltration. Indeed, the acute fivefold increase in circulating neutrophils upon re-exposure to HFD is striking and studies in mice and humans have shown that increases in circulating neutrophil counts causally link with increased risk for a cardiovascular event.^3^ It remains, however, unclear if this neutrophilia is sustained over time or occurs merely acutely after shift of the diet. Counts of circulating neutrophils are tightly regulated by several mechanisms including production in the BM, mobilization from the site of production, life span and clearance. Here, the authors identify higher numbers of neutrophil progenitors, namely GMPs, and increases in plasma C-X-C motif chemokine ligand (CXCL) 1 and CXCL2 arguing in favor of increased neutrophil production and facilitated mobilization, respectively. However, these data are not fully conclusive and open the way for additional future studies. As an example, time stamping of granulopoiesis by use of bromodeoxyuridine (BrdU) pulse labelling would allow to assess dynamics of production and release. In addition, the CXCL12-C-X-C chemokine receptor (CXCR) 4 axis is a key regulator of neutrophil retention in the BM and their return to sites of clearance. Future studies will need to reveal if this axis and the circulation time of neutrophils is altered upon HFD re-exposure. In this context it is also important to consider circadian fluctuations of neutrophil counts. In fact, neutrophil counts are highest in the activity phase of the mouse and much lower during day time. A recently published study showed that time-restricted feeding (TRF), a form of intermittent fasting, accelerates atherosclerosis by accentuating peaks of intrinsic neutrophil oscillations^4^ hereby enhancing arterial neutrophil infiltration. It would thus be interesting to study how re-exposure to HFD feeding impacts the circadian oscillations of neutrophil counts.

IL-1β is a master regulator of cardiovascular inflammation and its neutralization in humans was shown to drastically reduce cardiovascular risk in the canakinumab anti-inflammatory thrombosis outcomes study (CANTOs) trial. Yet, this study lends additional support to the multi-faceted actions of IL-1β in cardiovascular inflammation. In fact, IL-1β is found as a central mediator of hematopoietic stem and progenitor cell (HSPC) activation upon re-exposure to HFD and its neutralization blunts neutrophilia and lesion expansion. The latter may be due to effects at the BM level but also a consequence of inflammatory processes driven in the lesions such as NET-driven macrophage activation. Previous work has shown that IL-1β signaling accelerates cell division and myeloid differentiation of HSPCs.^5^ Chronic activation of this pathway limits the output of HSPCs and impairs the self-renewal capacity of HSPCs. Importantly, these effects are fully reversible upon withdrawal of IL-1β. Thus, it would be interesting to assess if re-exposure to HFD impairs HSPC function and if repeated re-exposure leads to HSPC exhaustion possibly augmenting host defense.

Altogether, findings by Lavillegrand and colleagues identify a novel mechanism of yoyo-diet induced cardiovascular inflammation and highlight the importance of altered granulopoiesis. Therapeutically, IL-1β or its receptor may stand out a therapeutic target of interference—alternatively, adhering to a continuous nutritional pattern may do the same trick.
